# A Case of an Elderly Woman Who Developed Corneal Perforation in the Clinical Course of Myeloperoxidase Positive Antineutrophil Cytoplasmic Antibody-Associated Vasculitis

**DOI:** 10.1155/2023/4246075

**Published:** 2023-08-26

**Authors:** Shuhei Kobayashi, Makoto Harada, Aiko Yamada, Yasuhiro Iesato, Koji Hashimoto, Yuji Kamijo

**Affiliations:** ^1^Department of Nephrology, Shinshu University School of Medicine, Matsumoto 390-8621, Japan; ^2^Department of Ophthalmology, Shinshu University School of Medicine, 3-1-1, Asahi, Matsumoto 390-8621, Japan

## Abstract

Antineutrophil cytoplasmic antibody- (ANCA-) associated vasculitis (AAV) is a systemic vasculitis characterized by ANCA positivity and categorized into three main types: microscopic polyangiitis, granulomatosis with polyangiitis, and eosinophilic granulomatous with polyangiitis. Although AAV leads to systemic organ injury, such as of the lungs, kidneys, nerves, and skin, patients with AAV sometimes develop ocular lesions. Here, we report the case of an elderly woman who had been treated for AAV for seven years. She developed scleritis and relapsed twice, with elevation of serum disease markers such as ANCA titer and C-reactive protein. After the decline of these markers due to treatment with additional medication, her scleritis relapsed again and caused a corneal ulcer, which resulted in perforation without obvious marker elevation. She did not present with any symptoms of organ injury, except for ocular lesions. She was treated with surgery, followed by methylprednisolone and rituximab therapy. Subsequently, her ocular lesions and symptoms improved, and she did not relapse. AAV can cause various ocular manifestations. Although C-reactive protein and ANCA titers are useful markers of disease activity and the relapse of AAV complications, including ocular lesions, these markers do not always increase at the time of worsening ocular lesions. Therefore, it is important for clinicians treating patients with AAV to pay careful attention to serum data and physical findings, including the eyes.

## 1. Introduction

Antineutrophil cytoplasmic antibody- (ANCA-) associated vasculitis (AAV) is characterized by ANCA positivity and leads to systemic organ injury, such as of the lungs, kidneys, nerves, and skin [1, 2]. AAV is categorized into three main types: microscopic polyangiitis (MPA), granulomatosis with polyangiitis (GPA), and eosinophilic granulomatous with polyangiitis (EGPA) [1, 2]. In addition, injury to other organs, such as head and neck manifestations, also develops, particularly in patients with GPA. The disease activity of ANCA-associated vasculitis is generally evaluated by organ injury using the Birmingham Vasculitis Activity Score (BVAS) [3] and serum biomarkers, such as C-reactive protein levels and ANCA titer [1, 4]. One of the clinical scores used to evaluate the severity of AAV is serum levels of C-reactive proteins [5].

Here, we report the case of a patient with recurrent ANCA-associated vasculitis, with a worsening of an ocular lesion but without elevation of C-reactive protein or ANCA titers during maintenance therapy. We present the clinical course of the patient and discuss the recurrence of AAV with ocular lesions and the relationship between worsening ocular lesions and serum disease activity markers of AAV.

## 2. Case Presentation

**Figure 1:**
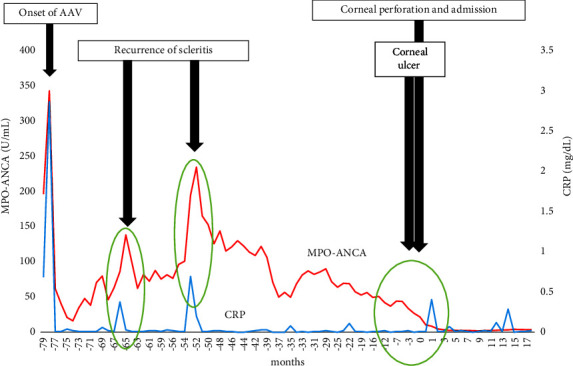
Clinical course of MPO-ANCA and C-reactive protein. During her clinical course, AAV and scleritis relapsed twice, at 65 months and 53 months before the current admission. At 65 and 53 months before the current admission, the MPO-ANCA titer was in the positive range and obviously increased, and C-reactive protein levels were also increased, although the C-reactive protein level was low, at less than 1.0 mg/dL (green circle). At the time of admission, C-reactive protein levels did not obviously increase (green circles). In addition, although the MPO-ANCA titer at the time of admission was positive, the titer was not obviously increased compared to the levels over the previous several months.

A 74-year-old woman developed bilateral hearing loss, scleritis, positivity for myeloperoxidase (MPO)-ANCA (the MPO-ANCA titer was >300 U/mL; the clinical course of MPO-ANCA, and C-reactive protein is presented in Figure 1), and hematuria (occult blood 2+ and red blood cells >30–49 per high-power field in sedimentation) seven years ago. A kidney biopsy revealed pauci-immune necrotizing crescentic glomerulonephritis, which resulted in a diagnosis of AAV. According to the European Medicines Agency algorithm of 2007 [6], she was found to have MPA due to primary systemic vasculitis revealed by the findings of kidney biopsy, MPO-ANCA positivity, and potential conditions that were ruled out. Autoimmune diseases associated with vasculitis were ruled out through negative results in antinuclear antibody and rheumatoid factor tests, as well as normal levels of complement 3, 4, and CH50. Furthermore, there were no specific skin lesions or joint symptoms indicative of autoimmune diseases.

In addition, the cause of scleritis was considered to be AAV, due to the other immunological disorders being ruled out. The patient's hearing loss was characterized by sudden bilateral sensorineural impairment over a span of two months. Magnetic resonance imaging of the cranial region, including the inner ear, did not reveal any specific abnormalities, indicating the absence of structural ear lesions. Furthermore, the bilateral and subacute nature of the hearing loss suggested a systemic inflammatory etiology. This led us to diagnose AAV as the most likely cause, which was confirmed as the patient fulfilled the relevant criteria for AAV. The clinical course of the ear lesion further aligned with the typical manifestations of AAV. Therefore, the symptoms of hearing loss and scleritis were considered to be caused by AAV. Her Birmingham Vasculitis Activity Score (BVAS) was found to be 14, consisting of scleritis, which added 2 points, sensorineural hearing loss, which added 6 points, and hematuria, which added 6 points.

**Figure 2:**
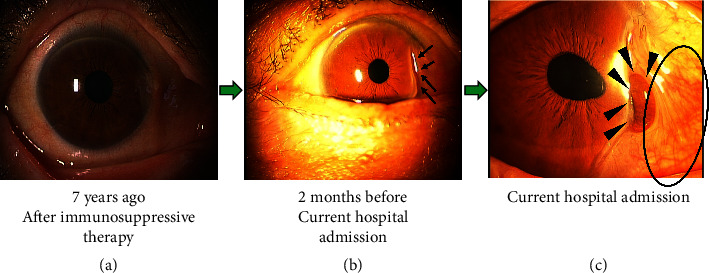
Right eye findings during the clinical course of the case. The clinical course of the findings in the right eye. After the initial immunosuppressive therapy at the time of the first AAV development, the scleritis improved. (a) The right eye findings two months before the current hospital admission. Corneal ulcers (arrows). (b) Right eye findings at the time of current hospital admission. Hyperemia in the sclera indicated scleritis (circle), corneal ulcer progression, and perforation (arrow heads) (c).

Treatment was started with methylprednisolone 500 mg pulse therapy and 30 mg daily oral prednisolone (PSL). Her hearing loss, scleritis, and urinary abnormalities gradually improved (Figure 2(a)) and the MPO-ANCA titer also decreased (Figure 1).

Azathioprine (AZA) was added as maintenance therapy against AAV, and the dose of oral PSL was gradually decreased to 5 mg/day. However, the patient presented with elevated transaminase levels, and it was suspected that liver injury had developed due to AZA. Therefore, AZA was changed to mizoribine (MZR) five years ago. During her clinical course, AAV and scleritis relapsed twice, at 65 months and 53 months before the current admission. At this time, the MPO-ANCA titer and C-reactive protein levels increased, although the C-reactive protein levels remained relatively low, at less than 1.0 mg/dL. With treatment for the relapsed scleritis, PSL increased, and scleritis improved. The patient did not present with any other organ injury or urinary abnormalities at the time of scleritis relapse. The PSL dose was then gradually reduced to 4 mg/day. Two months before her current hospital admission, she noticed right eye pain that gradually worsened. She was diagnosed with scleritis with a corneal ulcer (Figure 2(b)), and because the current ocular lesion was considered to be due to AAV relapse and infectious conditions were not detected, the PSL dose was increased to 30 mg/day. However, she had judged for herself and taken 8 mg/day even when the dosage was increased to 30 mg/day.

**Figure 3:**
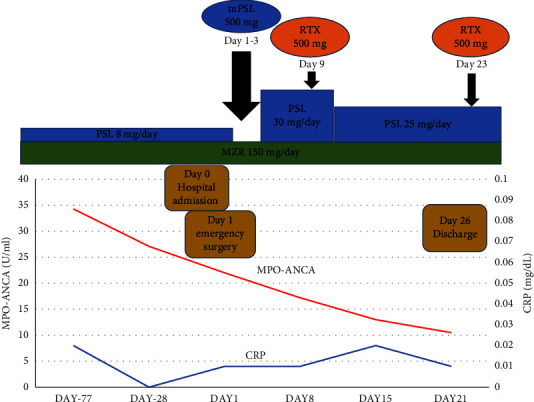
Clinical course of AAV treatment and MPO-ANCA and C-reactive protein titer changes. After the surgery, methylprednisolone 500 mg pulse therapy was administered for three days, and 30 mg/day of oral PSL was started. Rituximab (500 mg) was then administered twice. The MPO-ANCA titer decreased to 10.0 U/mL. The patient was discharged after the PSL dose was reduced to 20 mg/day.

Two months later, the ocular lesion worsened, and she was diagnosed with aggravation of the right scleritis with corneal perforation (Figure 2(c)). She was admitted to the ophthalmology department and underwent emergency surgery (keratoprosthesis). At the time of hospital admission, her serum C-reactive protein was 0.01 mg/dL, and her serum creatinine was 1.03 mg/dL. Urinary abnormalities were not detected, the MPO-ANCA titer was 22.0 U/mL (Table 1), and symptoms from other types of autoimmune diseases, except AAV, were not detected. Although her MPO-ANCA titer at admission was in the positive range, it did not obviously increase compared to the levels over the several previous months (Figures 1 and 3). Her BVAS was found to be 11, consisting of scleritis, which added 2 points, foggy vision, which added 3 points, and sudden visual loss, which added 6 points. Then, the following facts were supportive for considering that the current relapse of scleritis was due to AAV: she was diagnosed with AAV based on the criteria at the time of onset, her scleritis was also due to AAV at the time of onset, and the scleritis had also been improved by immunosuppressive therapy, along with other symptoms of AAV.

Immediately after keratoprosthesis surgery, methylprednisolone 500 mg pulse therapy was administered for three days, and 30 mg/day of oral PSL was restarted. In addition, glucocorticoid eye drops and antibiotic eye drops were also used. Furthermore, because the ocular lesion due to AAV relapsed again with PSL and MZR therapy, rituximab (500 mg) was administered twice. The MPO-ANCA titer decreased to 10.0 U/mL, and the patient was discharged after the PSL dose was reduced to 20 mg/day. The MPO-ANCA titer was negative two months after rituximab therapy, and the PSL dose was then reduced to 5 mg/day. She had no recurrence of eye or other organ symptoms after discharge. Mizoribine was continued during the current relapse and discontinued 4 months after the current relapse because she achieved remission and her ANCA titer became negative.

## 3. Discussion

In the current case, the ocular lesion due to AAV relapsed, and the patient did not present with obvious elevation of C-reactive protein or MPO-ANCA titer. In addition, the patient did not have any organ injuries due to AAV, except for an ocular lesion.

Among patients with AAV, the incidence of developing ocular lesions in patients with GPA is higher than that in patients with MPA or EGPA [1, 7]. A previous report suggested that 16% of patients with AAV or 39–58% of those with GPA presented with ocular lesions [8–11]. The most common ocular manifestations are eye pain and loss of visual acuity [9]. In addition, alterations in visual acuity, hyperemia, visual field impairment, and lacrimation have been observed [12], as have scleritis (22–29%), episcleritis (21–29%), orbital disease (18–21%), lacrimal duct stenosis (10%), and uveitis (9%) [9, 10]. Conjunctivitis, keratitis, dacryoadenitis, optic neuritis, retinal detachment, and orbital bone destruction have also been reported. Thus, clinicians need to be aware that AAV can cause various eye symptoms and lesions. In addition, although more than 40% of patients with GPA with ocular lesions have been treated with immunosuppressive therapy in addition to glucocorticoids, their ocular lesions were often refractory and/or recurred (22.2% of patients with GPA with ocular lesions experienced recurrence), resulting in the additional administration of rituximab treatment [10]. Therefore, patients whose AAV is complicated by ocular lesions may need to be treated with glucocorticoids and immunosuppressive agents, such as rituximab. Concerning the risk factors for ocular lesions in patients with AAV, positivity for proteinase 3-antineutrophil cytoplasmic antibody (PR3-ANCA) has been reported to be an independent risk factor [11]. The presented case manifested as scleritis, leading to a subsequent corneal perforation. Scleritis, as distinct from episcleritis, can give rise to significant ocular morbidity and typically presents with profound, penetrating pain and sensitivity to light [13]. The condition is characterized by the presence of dilated blood vessels in a network-like appearance. In addition, the risk of scleral thinning and perforation can be further exacerbated by secondary infections. Effective management of scleritis necessitates systemic immunosuppression to address the underlying disease, alongside an aggressive approach to ocular care. Severe cases of scleritis often require topical antibiotic therapy to prevent bacterial infections. In certain instances, scleral patch grafts may be considered as a suitable treatment modality.

Serum ANCA titers and C-reactive protein levels are useful for evaluating disease activity in patients with AAV, and C-reactive protein levels generally correlate with disease progression [1, 5, 14]. Jayne et al. reported that 83% of patients with recurrent AAV tested positive for ANCA during a follow-up period, among 60 patients with AAV after clinical remission [15]. In addition, 57% of patients had elevated ANCA titers prior to relapse. However, in our patient, although C-reactive protein and MPO-ANCA titers at the time of previous relapse of scleritis were obviously increased, they did not increase at the time of recent scleritis relapse with corneal perforation. We searched for previous cases of patients with AAV with ocular lesions and extracted case reports that described C-reactive protein and ANCA titers [12, 16–27]. We then investigated the relationship between patients with AAV with ocular lesions and C-reactive protein levels, as well as ANCA titers (Table 2).

We obtained information about the clinical course and data on C-reactive protein and ANCA in 19 adult patients with AAV (including the current patient) complicated with ocular lesions. Information on age, sex, type of ANCA, ocular lesions, other organ involvement, levels of C-reactive protein and ANCA, and treatment is presented in Table 2. The patients were 19 to 74 years old, with a male : female ratio of 9 : 10. Positivity for MPO-ANCA was detected in three patients, positivity for PR3-ANCA in six, positivity for C-ANCA in seven, and positivity for P-ANCA in four (one patient was positive for both C and P-ANCA). Although various ocular manifestations were observed, the most common was scleritis. Elevation of C-reactive protein levels was observed in 13 patients, while six did not show elevation. Thus, it is not rare for ocular lesions to flare up without elevation of C-reactive protein levels in patients with AAV.

AAV can cause various ocular manifestations. C-reactive protein and ANCA titers are useful markers of disease activity and relapse of AAV complications, including ocular lesions; however, these markers do not always increase at the time of ocular lesion worsening. Therefore, it is important for clinicians who treat patients with AAV to pay careful attention to not only serum data but also physical findings, including findings related to the eyes.

## Figures and Tables

**Table 1:** Main clinical data of the current case at current hospital admission.

Urinalysis			Total protein	6.8	g/dL
Protein	—		Albumin	4.6	g/dL
Hematuria	—		BUN	20.6	mg/dL
	<1	/HPF	Cr	1.03	mg/dL
NAG	1.3	U/L	UA	4.9	mg/dL
*β*2-MG	24	*μ*g/L	Na	141	mEq/L
			K	4.3	mEq/L

Blood analysis			Cl	104	mEq/L
WBC	6290	/*μ*L	AST	23	U/L
Neut	69.7	%	ALT	12	U/L
Lym	23.4	%	LDH	175	U/L
Mono	4.5	%	ALP	165	U/L
Eos	4.5	%	*γ*GT	13	U/L
Baso	1.6	%	T.Bil	0.8	mg/dL
Hb	15.0	g/dl	CRP	0.01	mg/dL
Plt	23.3 × 10^4^	/*μ*L	MPO-ANCA	22.0	U/mL

ALP, alkaline phosphatase; ALT, alanine aminotransferase; AST, aspartate aminotransferase; *β*2-MG, *β*2-microglobulin; BUN, blood urea nitrogen; Cl, chloride; Cr, creatinine; CRP, C-reactive protein; *γ*GT, gamma-glutamyl transpeptidase; Hb, hemoglobin; K, potassium; LDH, lactate dehydrogenase; MPO-ANCA, myeloperoxidase antineutrophil cytoplasmic antibody; Na, sodium; NAG, N-acetyl-*β*-D-glucosaminidase; Plt, platelet; T.Bil, total bilirubin; UA, uric acid; WBC, white blood cell count.

**Table 2:** Previously reported AAV cases with ocular lesions (CRP and ANCA data available).

	Age	Sex	ANCA type	ANCA titer	CRP (mg/dL)	Ocular manifestation	Organ involvement	Treatment
[16]	52	M	P	Positive	16.7	Scleritis	Neuropathy	PSL, mPSL, CY, MTX
[16]	59	F	P	219 EU	>10	Scleritis	Kidney	PSL
[16]	42	M	P	>640 EU	3.1	Scleritis	Lung	PSL, mPSL, CY
[21]	73	F	MPO	235 U/mL	10.4	Bilateral orbital apex syndrome	ENT	PSL, mPSL, MTX
[22]	69	F	MPO	124 RU/mL	0.4	Scleritis	ENT	PSL, mPSL, RTX, AZA
Current case	74	F	MPO	22.0 U/mL	0.01	Scleritis, cornea ulcer, perforation	—	PSL, mPSL, MZR, RTX
[17]	54	M	C	Positive	Elevated	Necrotizing scleritis, uveitis	ENT	PSL, mPSL, MTX
[17]	56	M	C	Positive	16.8	Necrotizing scleritis, ulcerative keratitis	—	PSL, mPSL, CY
[17]	50	F	C	Positive	13.7	Necrotizing scleritis, ulcerative keratitis, cornea ulcer	Lung	PSL, mPSL, CY, MTX
[17]	30	M	C	Positive	43.0	Necrotizing scleritis, ulcerative keratitis	ENT, lung, kidney	PSL, CY, IVCY
[12]	37	M	C	Positive	7.7	Scleritis, corneal perforation conjunctivitis	ENT, lung	IVCY, CY, mPSL
[23]	38	M	C	Positive	7.2	Conjunctivitis, episcleritis, anterior uveitis	Kidney	PSL, mPSL, RTX, PE
[24]	53	F	PR3	175 IU/mL	1.2	Episcleritis	Kidney, muscle, joint	PSL, CY, AZA
[19]	67	M	PR3	23 EU/mL	0.2	Scleritis	ENT	PSL, mPSL, IVCY, RTX
[25]	44	F	PR3	5.9 U/mL	0.12	Scleritis	ENT, skin	PSL, mPSL
[26]	73	F	PR3	725 U/mL	0.4	Scleritis	Kidney, lung	PSL, RTX
[18]	40	F	PR3	195 AU/mL	6.6	Ulcerative keratitis	ENT, lung	PSL, CY
[20]	19	F	PR3	Positive	11.0	Necrotizing scleritis, ulcerative keratitis	ENT	PSL, AZA, RTX, MTX
[27]	74	M	P and C	28 EU/mL and 23 EU/mL	0.05	Scleritis	—	PSL, mPSL

AAV: antineutrophil cytoplasmic antibody-associated vasculitis, ANCA: antineutrophil cytoplasmic antibody, AZA: azathioprine, CRP: C-reactive protein, CY: cyclophosphamide, ENT: ear, nose, and throat, ESR: erythrocyte sediment rate, F: female, IVCY: intravenous cyclophosphamide, M: male, MPO: myeloperoxidase, mPSL: methylprednisolone, MTX: methotrexate, PE: plasma exchange, PR3: proteinase 3, PSL: prednisolone, RTX: rituximab.

## Data Availability

The data used to support the findings of this case report are available from the corresponding author upon request.
